# Biopolymer Gels as a Cleaning System for Differently Featured Wooden Surfaces

**DOI:** 10.3390/polym15010036

**Published:** 2022-12-22

**Authors:** Chaehoon Lee, Francesca Di Turo, Barbara Vigani, Maduka L. Weththimuni, Silvia Rossi, Fabio Beltram, Pasqualantonio Pingue, Maurizio Licchelli, Marco Malagodi, Giacomo Fiocco, Francesca Volpi

**Affiliations:** 1 Department of Chemistry, University of Pavia, 27100 Pavia, Italy; 2 Arvedi Laboratory of Non-Invasive Diagnostics, CISRiC, University of Pavia, 26100 Cremona, Italy; 3 National Enterprise for nanoScience and nanoTechnology (NEST), Scuola Normale Superiore, Piazza dei Cavalieri 7, 56126 Pisa, Italy; 4 Department of Drug Sciences, University of Pavia, 27100 Pavia, Italy; 5 Department of Musicology and Cultural Heritage, University of Pavia, 26100 Cremona, Italy

**Keywords:** sodium alginate, konjac glucomannan, gel cleaning, profilometer, X-ray fluorescence, texture analysis, green gel

## Abstract

The cleaning of some wooden artefacts can be challenging due to peculiar surface roughness and/or particular finishing treatments that favour the deposition of dirt and contaminants. The most common cleaning system used by conservators is agar gel, characterized by its rigidity and brittleness, which challenges the cleaning of rough and irregular surfaces typical of most wooden artefacts. In this work, alginate crosslinked with calcium (CA) and konjac glucomannan crosslinked with borax (KGB) gels were proposed to solve this issue. They were prepared and applied to smooth- and rough-surfaced mock-ups replicating wooden musical instruments’ surfaces that had been subsequently covered by artificial soiling and sweat contaminants. The mechanical properties of CA and KGB gels, including their stability over a 60-day storage time, were evaluated by a texture analyzer, while cleaning efficacy was analytically evaluated by non-invasive X-ray fluorescence mapping and profilometric investigation. CA gel appeared to have a higher tensile strength and elongation at break. KGB gel was shown to be soft and resilient, indicating its suitability for cleaning rough surfaces. After repeating the cleaning application three times on the rough-surfaced mock-ups, both the CA and KGB gels were shown to have cleaning efficacy. The results obtained with CA and KGB were compared with those from the Agar application.

## 1. Introduction

Cultural artworks are a valuable heritage that conservators, restorers, researchers, and other stakeholders strive to preserve. Nevertheless, long-term storage in an indoor environment and improper care frequently lead to an accumulation of diverse foreign matters, such as dust and contaminants that could interact with and alter artefact surfaces [1]. Therefore, over the years, the most suitable cleaning procedures were employed for the optimal preservation of artefacts, preventing physical and chemical deterioration in most cases. Different cleaning approaches, such as gentle brushing, vacuuming, and swab rolling with aqueous or organic solvents, are commonly used, depending on the surface condition, the type of contaminants, and the material to be treated (e.g., paper, stone, metal) [2,3,4]. However, these cleaning procedures may induce colour changes or mechanical stress on fragile surfaces, as well as the leaching and swelling of sensitive layers underneath the cleaned surface [5,6,7]. Conversely, the use of gels could be a suitable alternative to prevent damage to artefacts since the three-dimensional cross-linked polymer network, chemically or physically bonded [8], controls the solvent’s swelling and spreading degree. Due to characteristics such as controlled solvent release, softness, and ease of application, gel cleaning became the preferred method for cultural heritage materials [9,10,11,12,13,14,15,16]. Among these, the use of biopolymers and green gels was particularly encouraged as they are eco-sustainable, biodegradable, non-hazardous, and consequently safe for the conservators’ work environment [9,17,18,19,20].

**Figure 1:**
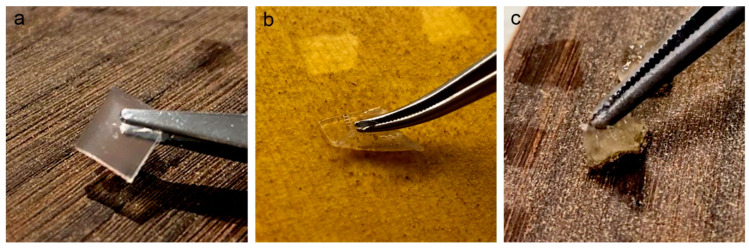
Images of application of Agar gel on soiled-EAM (**a**), CA gel on soiled-WM (**b**), and KGB gel on soiled-EAM (**c**).

Natural polysaccharide-based gels are widely used for cleaning heritage materials due to their tunable rheological properties and mechanical behaviours [21,22], their easy large-scale production from sustainable sources, and their low price. The most used polysaccharide gels are agar and gellan gum [20,23,24], both physically rigid gels that are commonly applied for cleaning paper-based materials and paintings. However, they may have poor cleaning efficacy on rough surfaces due to their rigidity and brittleness and leaving gel residue on treated surfaces (Figure 1a) [6,25]. To overcome these limitations, alginate and konjac glucomannan were chosen as suitable alternatives to agar due to their promising texture properties and solvent retention capabilities. Alginate is a polysaccharide derived from brown algae, and konjac is derived from the tubers of Amorphophallus konjac. Both materials are biocompatible, biodegradable, non-toxic, and widely used in the biomedical and pharmaceutical fields, as well as in the food industry [26,27,28,29].

In the previous study [30], alginate and konjac natural polysaccharides were prepared with different crosslinkers and loaded with a biodegradable surfactant to obtain the best conditions for cleaning. The ionically crosslinked calcium alginate (CA) appeared to have high solvent retention capability and high surface contact for smooth surfaces due to its film form. Whilst borate-crosslinked konjac (KGB) appeared as a soft form gel with high solvent loading capability and when handling, it exhibited an elastic texture, suitable for cleaning irregular and rough surfaces. Consequently, CA and KGB gels are biocompatible and effective in cleaning regular or irregular wooden surfaces. Moreover, they demonstrated a good ability to retain the solvent fraction within the gel network, avoiding excessive spreading on the surface, and offering a promising and affordable alternative in the field of cultural heritage conservation. The successful results obtained so far encouraged further investigations of their mechanical properties (i.e., tensile strength, hardness, and elasticity) and a detailed examination of their cleaning efficacy.

Hence, this research is aimed at (i) understanding the mechanical properties (i.e., tensile strength, hardness, and elasticity) of CA and KGB gels, as well as their stability over a 60-day storage time, and (ii) providing a detailed analytical evaluation at the microscale level of the cleaning efficacy on both flat and smooth Western varnished surfaces and rough and irregular surfaces replicating Asian musical instruments.

Two different sets of wooden mock-ups inspired by the surfaces of historical musical instruments of the Western area (classical bowed string quartet) and East Asian traditional music (primarily the zither instrument) were prepared. For the Western mock-up (WM), a smooth surface finished with an oil-based varnish was prepared [31,32,33]. For the East-Asian mock-up (EAM), a rough surface treated with the so-called ‘Nakdong technique,’ a traditional scorching application using a hot iron held on the surface of the wood, was prepared [34,35]. Both sets of mock-ups were then covered with two different synthetic contaminants (soiling and sweat) and cleaned with CA and KGB gel. The cleaning efficacy of the gels was consequently revealed using a stereomicroscope, X-ray fluorescence (XRF), and profilometric analysis. The evaluation of the mechanical properties and the stability over time were performed through the texture tests. Depending on the gel form, two different tests were considered: a tensile test for film-form and a uniaxial compressive test for soft form gels. Depending on the type of test used (tensile or compressive test), it was possible to evaluate different gel mechanical properties. Hardness and Young’s modulus were calculated for soft form gels, while tensile strength and elongation at break were calculated for film-form gels. In addition, each fresh and aged gel was examined to investigate its physical stability.

## 2. Materials and Methods

### 2.1. Mock-Ups

For Western mock-ups (WM), in order to reproduce the coating system generally identified on the Cremonese bowed string instruments [36,37], spruce wood samples of 10 cm × 10 cm × 1 cm (longitudinal × radial × tangential directions) were smoothed and cleaned to remove the dust from the surface similarly to real instruments. After cleaning, pore filling was performed with rabbit glue (10% *w*/*w* in water), with the ground layer being applied using a brush to create a regular surface before applying varnish. After that, two applications of an oil-based varnish composed of linseed oil and colophony (1:1 ratio) were applied by brush and dried under UV light for 30 h, as explained in the literature [38].

For the East Asian mock-ups (EAM), paulownia wood of 10 cm × 10 cm × 1 cm (longitudinal × radial × tangential directions) was treated with the Nakdong technique. Inside the hot kiln stove, a traditional iron tool specifically used for this treatment, customized by a blacksmith, was heated until red-hot. Afterward, the red-hot iron tool was pressed onto the wood in one stroke for 1–3 s [35].

On the prepared WM and EAM mock-ups, a synthetic soiling mixture (dry portion *w*/*w*: burnt sienna pigment 0.5%, gelatine 10.4%, soluble starch 10.4%, Portland type I cement 20.5%, silica 1.9%, lime 16.7%, kaolin 18.6%, and peat moss 20.8%; and wet portion *v*/*v*: mineral oil in chloroform 4.5%) and standardized synthetic sweat in accordance with DIN ISO 9022-12 were dispersed, as reported in Lee 2022 [30]. For the contaminants, all the soiling components were purchased from Bresciani Srl (Milan, Italy), and synthetic sweat was purchased from Synthetic Urine e.K. (Eberdingen, Germany).

### 2.2. Sodium Alginate and Konjac Glucomannan Gels Preparation

Sodium alginate, medium viscosity (A2033), and Mw ~ 80,000 to 120,000 g/mol, was purchased from Sigma Aldrich (St. Louis, MO, USA). Konjac glucomannan, purity ≥ 95%, and Mw ~ 960,000 g/mol was provided by Hubei Yizhi Konjac Co., Ltd. (Changyang, Hubei, China). Calcium chloride and borax were also purchased from Sigma Aldrich. For CA gel, sodium alginate 2% *w*/*v* was stirred in distilled water for 30 min at room temperature until completely dispersed, and was then poured into the petri dish until it reached a thickness of 2 mm. To cast the gel, a CaCl_2_ 2% *w/v* solution in distilled water was poured and maintained in immersion for 15 min. In this way, the CA thin film-form gel was formed, characterized by a homogeneous surface and a thickness of less than 2 mm (Figure 1). For KGB gel, konjac glucomannan 2% *w/v* was dispersed in distilled water at 50 °C, and while stirring, 0.1 g of borax was added to 10 mL konjac glucomannan dispersion. The formed KGB was left at room temperature for 30 min to stabilize the mixture, and then it was bathed at 90 ± 10 °C for 20 min in deionized water. The immersed KGB gel was taken out, cooled, and dried with Japanese paper until the water on the surface dried. The flow chart for the preparation of CA and KGB gels is presented in Figure S1 in the Supplementary Information. The KGB appeared as a soft form gel, shown in Figure 1, with pliable and elastic features.

### 2.3. Cleaning Procedure

The prepared CA and KGB cleaning gels were loaded with the non-ionic and biodegradable surfactant Ecosurf TM EH-9 (EH-9, Sigma Aldrich, St. Louis, MO, USA) by soaking the gels for 24 h and 5 h, respectively, in a 2% (*v/v*) surfactant water solution. The soaking time was decided according to the fully swollen states of the two gels reported in Lee 2022 [30]. The cleaning was performed for 5 min and repeated up to three times (CA_1—CA_3, KGB_1—KGB_3, and Agar_1—Agar_3) on the mock-up surfaces where synthetic soiling and sweat were dispersed (soiled-WM, soiled-EAM, sweat-WM, and sweat-EAM). During the cleaning application, the surfactant, which is confined within the retentive network of the gels, reaches the contaminant on the mock-up surface, softening it. Then the sweat or dust is gently removed by removing the gel from the surface.

### 2.4. Mechanical Properties of Fresh and Aged Gels

The mechanical properties of the gels were evaluated by stretching (tensile test) or by compression (compressive test) [39,40,41,42]. During the tensile test, films were uniaxially stretched until failure to measure their tensile strength and elongation at break (Figure S2). The compressive test involves gels being stressed by uniaxial forces perpendicular to the sample surface, which allowed the study of gel hardness and elasticity. According to gel properties and application, texture analyses can be carried out by setting different experimental parameters to achieve the information in a reproducible manner [43]. To investigate long-term physical stability, both fresh gels (tested immediately after preparation) and aged gels (stored for 60 days at 4 °C) were studied [44]. The 60-day storage period was selected as a reasonable length of time for conservators to spend on cleaning treatments.

Since CA and KGB gels were in film and soft forms, respectively, both mechanical tests were used. CA gel mechanical properties were assessed through a tensile test by means of a TA.XT plus Texture Analyzer (Stable Micro Systems, Godalming, United Kingdom), equipped with a 5 kg load cell. Each sample of gel, respectively, with a 2 mm thickness in the film form (1 cm × 3 cm), was clamped on an A/TG tensile grips probe, and an initial distance of 1 cm between the grips was set. The upper grip was raised at a constant speed of 2 mm/min for a distance of 50 mm. Before testing, the film thickness was measured using a Sicutool 3955G-50 (Milan, Italy) apparatus. Tensile strength and elongation at break were calculated; at least three measurements were performed for each film.

KGB gel was prepared in cylindrical moulds of 30 mm × 30 mm (diameter × height) and stored at 4 °C before acquiring the compression measurement. The test was accessed by a TA.XT plus Texture Analyzer, equipped with a 5 kg load cell and a P/10 measuring system consisting of a cylindrical probe with a diameter of 10 mm. The probe was lowered with a test speed equal to 1.00 mm/s to determine a 70% sample deformation. Two measurements were carried out for each gel. Compressive stress-strain curves were produced, and the following parameters were considered: (i) hardness as the maximum compressive force (Fmax) per unit area required for sample destructuring; (ii) Young’s modulus (YM) calculated as the slope of the tangent at the first part of the compressive stress–strain curve (<5% of strain) to investigate the ability of each gel to withstand changes in length when subjected to compression.

Experimental data were subjected to statistical analysis, carried out through the statistical package Statgraphics 5.0 (Statistical Graphics Corporation, Rockville, Maryland, USA). In particular, a one-way ANOVA was carried out, followed by a Multiple Range Test.

### 2.5. X-ray Fluorescence Analysis

X-ray fluorescence (XRF) represents one of the most used elemental analysis in the field of archaeometry and diagnostics of cultural heritage [45,46,47], thanks to its quickness, clarity, and non-invasiveness.

XRF measurements were carried out using the energy-dispersive portable XG Lab—Bruker (Bruker Optics, Billerica, MA, USA) ELIO spectrometer with a Rh anode and a 1.2 mm collimator. XRF maps were carried out at a tube voltage of 40 kV and a tube current of 40 µA, with a measurement time of 3 s and a step size of 1 mm. Data were collected and elaborated with Elio 1.6.0.29 software. Based on the previous XRF investigation on cleaned and uncleaned areas [30], calcium (Ca) was selected as the marker element for the soiling mixture (containing cement) in soiled-WM and soiled-EAM, and chlorine (Cl) as the marker for the synthetic sweat (containing sodium chloride and ammonium chloride [48]) in the sweat-WM and -EAM. The counts per pixel, relative to marker elements, were calculated with pyMCA software version 5. 6. 7., ROI imaging tool on areas of 4 pixels (2 × 2 in pixel). For the evaluation of the cleaning efficacy, mean values and standard deviations were calculated by the following equation [49]:

$$Cleaning\;efficacy\;\left(\%\right)=1-\left(\frac{counts\;per\;pixel\;of\;cleaned\;area}{counts\;per\;pixel\;of\;uncleaned\;area}\right)\times 100\;\%$$

### 2.6. Surface Topography Evaluation

To visually overview the cleaning actions on the contaminated mock-ups, an Olympus stereomicroscope (Olympus, Tokyo, Japan) was used with an Olympus HD DP73 camera. Images were acquired using Stream Essentials 2.1 software.

To investigate the complex surface of the mock-ups, 3D profilometric maps and roughness data were acquired by means of a non-contact 3D Taylor Hobson profilometer (Taylor Hobson Ltd., PO Box 36, 2 New Star Road, Leicester, LE4 9JQ, United Kingdom).

Optical profiling is a non-contact, interferometric-based method used for the characterization of surface topography and is commonly applied in several fields, from cultural heritage to engineering and medical applications [50,51,52]. In particular, in the case of cultural heritage materials, the profilometer provided relevant information on smooth surfaces with roughness in the nanometer range in order to detect cracks and defects [53]. Optical profiling gives a wide range of roughness parameters, calculated according to specific ISO standards, which describe a given surface [54,55] according to the specific needs of the material and research. In addition, 3D maps of the surface can be obtained. The analyses were performed using 20× magnification with 4.7 mm of working distance, covering a surface analysis of 0.8 mm on cleaned areas. Profilometric measurements were performed on samples with a non-reflective surface to obtain a reliable 3D reconstruction of the surface: soiled-WM, soiled-EAM, and sweat-EAM. Due to the highly reflective surface, no profilometric measurements, including 3D maps and roughness parameter calculations, were performed on sweat-WM. Five measurements on each cleaned area were performed in order to have an indication of the original surface after the cleaning procedure. The roughness parameters and the 3D maps were obtained from the surface analysis and elaborated with the software TalyMap Gold 6.2. Maps. Roughness parameters obtained from 3D maps were calculated as defined in ISO 25178.

The amplitude parameters were selected in this study to describe the surface of the mock-ups before and after cleaning: arithmetic mean height (Sa), skewness (Ssk), and a maximum depth of valleys (Sv) [50,54]. This article excludes the mathematical explanation of these parameters, referring to specific texts in the literature [54,55,56,57]. Sa is the most used roughness parameter for quality control. It is defined as the average of the absolute deviation of the roughness irregularities from the mean line and provides a good general description of height variations, although it is not sensitive to small variations in the sample. Sv is defined as the maximum depth of the profile below the mean line within the assessment length. Ssk is used to measure the symmetry of the profile about a mean line, meaning this parameter is sensitive to occasional deep valleys or high peaks. A symmetrical height distribution (number of valleys equal to the number of heights) has zero skewness. Profiles with a majority of valleys have negative skewness. Profiles with valleys filled in or high peaks have positive skewness. The value of Ssk depends on whether the bulk of the material sample is above (negative Ssk) or below (positive Ssk) the mean line.

## 3. Results and Discussion

### 3.1. Stability of Gels: Mechanical Properties of Fresh and Aged Gels

**Figure 2:**
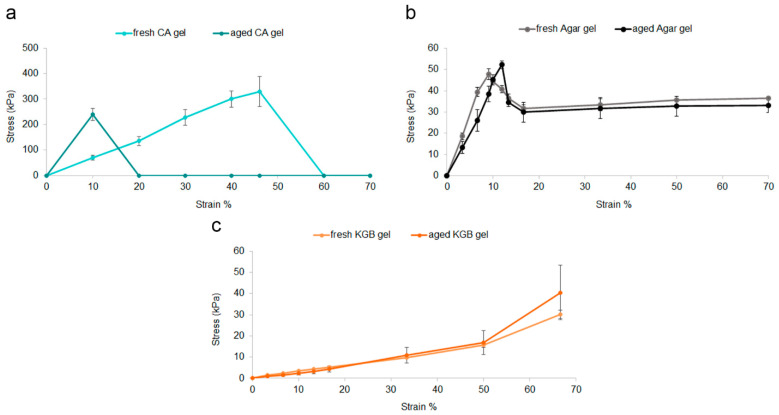
Stress–strain curves of fresh and aged Agar gel (**a**), CA gel (**b**), and KGB gel (**c**).

Different mechanical tests were performed on alginate gel crosslinked with calcium cations (CA) and konjac gel linked with borax (KGB), which were prepared in the film and soft forms, respectively. Measurements of Agar gels in thin and thick forms were added to this study for comparison purposes. Comparing fresh-CA and -Agar gels, CA was characterized by statistically significant higher tensile strength and elongation at break values (Table 1). Agar gel appeared very fragile and broke easily without stretching, while CA gel plastically deformed up to the breaking point. In Figure 2, the stress–strain curve describing the plastic behaviour of fresh-CA gel during the tensile test can be observed; it was not possible to plot the tensile stress versus strain % values of thin form Agar gel due to its mechanical weakness.

After 7 days of storage at 4 °C, microorganisms were already observed on the thin-form Agar gel, and after 60 days, it appeared dry. As a result, no measurements were performed on the aged-Agar gel as it could affect the accuracy of this assay. By comparing fresh- and aged-CA gels, a significant decrease in elongation at break appeared after 60 days (Figure 2b, Table 1). Although CA gel becomes less mechanically resistant with age, its mechanical properties are significantly higher than those of Agar fresh gel, representing an advantage for conservators who may use CA gel for cleaning even after 60 days.

Comparing fresh-KGB gel with fresh-Agar when subjected to a uniaxial compressive force, the latter showed a hardness of 47 kPa and a Young’s modulus of 556 kPa, which are significantly higher than KGB values (Table 2). These results of fresh-Agar gel were in accordance with other studies which described Agar as a rigid gel [23,24,25]. High hardness values and Young’s modulus correspond to the high stiffness of the gel, whereas low values correspond to greater elasticity and softness. Lower hardness values and Young’s modulus, therefore, suggest that KGB gel could be more suitable for cleaning rough surfaces.

In KGB gel, the hydroxyl groups of the konjac chains crosslink with the borax ions, creating physical connections that confer a gel-like behaviour, as the rheological tests demonstrated on a comparable formulation of konjac–borax gel [58]. In Figure 2a,c, the stress–strain curves demonstrated higher flexibility of KGB compared to Agar gel. After a 60-day storage at 4 °C, no statistically significant differences were found in both hardness and Young’s modulus between fresh and aged gels, regardless of the gel composition (Table 2). The Young’s modulus values assessed for aged gels still ensure their elastic properties can be effectively used for cleaning purposes even after 60 days.

### 3.2. Evaluation of the Gels Cleaning Efficacy by XRF Mapping

**Figure 3:**
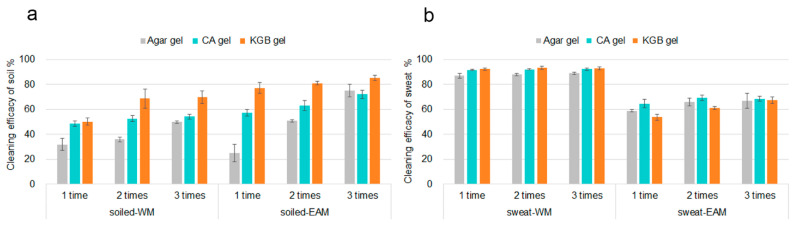
Cleaning efficacy values obtained on soiled-WM and soiled-EAM (**a**) and on sweat-WM and sweat-EAM (**b**). The percentage of cleaning efficacy was calculated based on the counts of Ca for soiled mock-ups and Cl for sweated ones. Agar, CA, and KGB gels were applied 1 to 3 times on both soiled- and sweat-contaminated mock-ups.

The cleaning efficacy of CA, KGB, and Agar gels applied one time (CA_1, KGB_1, and Agar_1) up to three times (CA_3, KGB_3, and Agar_3) was calculated using XRF mapping data, as explained in Section 2.5 (Figure 3).

On both soiled-WM and soiled-EAM, KGB_1 to KGB_3 demonstrated a higher percentage of cleaning efficacy than CA_1 to CA_3 (Figure 3a). By repeating the gel application, the cleaning efficacy of both CA and KGB gels increased, especially for KGB applied to soiled-WM, where the cleaning efficacy increased from KGB_1 to KGB_2 and remained unchanged until KGB_3. Repeated cleaning applications with Agar showed a significant increase in cleaning efficacy from Agar_1 to Agar_2, and from Agar_2 to Agar_3. After three applications on soiled-WM and soiled-EAM CA and Agar gel showed similar cleaning effectiveness, although less efficient than KGB gel.

The removal of sweat was better completed on the smooth surface of sweat-WM than sweat-EAM, as it appeared to have over 90% cleaning efficacy by both CA and KGB gel (Figure 3b). Additionally, sweat-WM, CA, KGB, and Agar gels showed excellent and comparable cleaning efficacy after a single application, which also resulted in the second and third cleaning repetitions. Conversely, on sweat-EAM, the cleaning effectiveness measured for CA and KGB gels increased after three cleaning applications, likewise for the reference Agar gel. This result was expected as sweat marks were observed with the stereomicroscope on the hydrophobic varnished surface of the WM but not on the EAM [30], possibly because the hydrophilic and porous structure of the paulownia wood used for the EAM may have adsorbed the contaminant. The result suggests that when gel cleaning is performed on hydrophilic surfaces contaminated with sweat, such as sweat-EAM, repeated cleaning trials should be recommended. Conversely, on hydrophobic surfaces such as the varnished WM, a single application could be enough to achieve satisfactory removal efficacy.

### 3.3. Surface Topography Evaluation

In order to deepen this study of the surface features in relation to the XRF results described in Section 3.2, careful observations with a stereomicroscope and profilometry were conducted on the areas selected for cleaning, documenting the subsequent steps of application of CA and KGB gels. In addition, Agar gel was tested in the same conditions for comparison. In detail, the cleaning effectiveness after repeated applications (from 1 to 3) was highlighted by profilometric 3D maps and the related roughness parameters, namely Sa, Sv, and Ssk, which, according to Janus 2010 and Rodriguez 2009 [51,59], can indicate the quality of polishing. Some additional figures, including 3D profilometric maps and stereomicroscope images, are presented in the Supplementary Information (Figure S3 and Figure S4).

**Figure 4:**
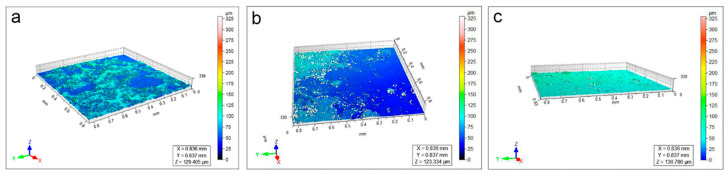
3D heat maps of soiled-WM (**a**) cleaned by CA_3 (**b**) and KGB_3 (**c**). The colour range represents the different heights (in microns) of the details present on the sample surface.

**Figure 5:**
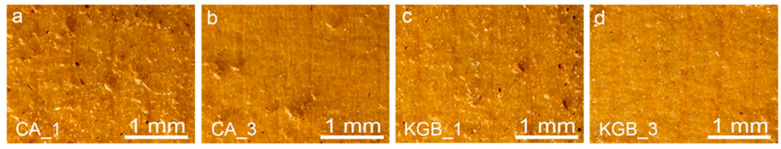
Images by stereomicroscope of the soiled-WM cleaned by CA gel and KGB gel at different application times CA_1 (**a**), CA_3 (**b**), KGB_1 (**c**), and KGB_3 (**d**).

**Figure 6:**
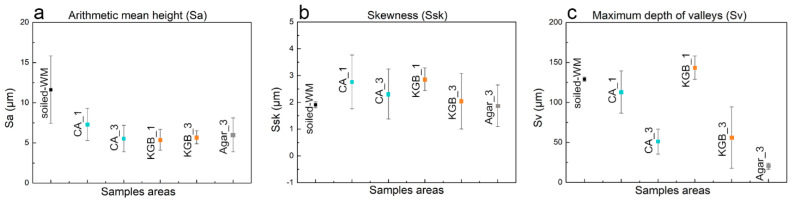
Considered roughness values of Sa (**a**), Ssk (**b**), and Sv (**c**) on the different areas of soiled−WM cleaned by different gels and application time CA_1, CA_3, KGB_1, KGB_3, and Agar_3.

Regarding soiled-WM, it is clear that the high roughness of the surface is strictly related to the presence of deposits (Figure 4a). After cleaning, the particles decrease in concentration due to subsequent applications of CA and KGB (from 1 to 3), with a consequent reduction in the surface roughness documented by 3D maps (Figure 4 and Figure S4a,b) and stereomicroscope (Figure 5a–d and Figure S3). The roughness parameters obtained by the profilometric measurements confirm this trend visible in Figure 6: the values of Sa (a) and Ssk (b) are comparable between CA and KGB, as well as for the Agar control. In this mock-up, Sv (c) seems to describe very well the surface roughness reduction through the cleaning applications: both the values related to CA and KGB decrease as a consequence of repeated applications. The Sv related to Agar is displayed here at a slightly lower value.

**Figure 7:**
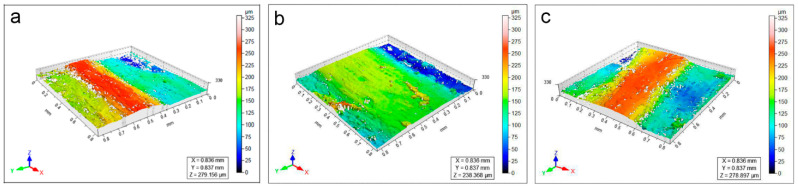
3D heat maps of soiled-EAM (**a**) cleaned by CA_3 (**b**) and KGB_3 (**c**). The colour range represents the different heights (in microns) of the details present on the sample surface.

**Figure 8:**
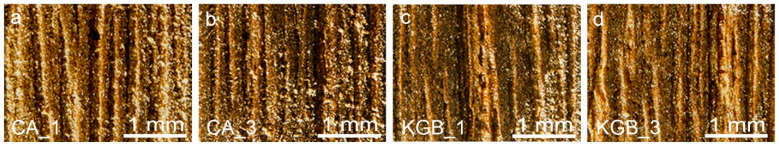
Images by stereomicroscope of the soiled-EAM cleaned by CA gel and KGB gel at different application times CA_1 (**a**), CA_3 (**b**), KGB_1 (**c**), and KGB_3 (**d**).

**Figure 9:**
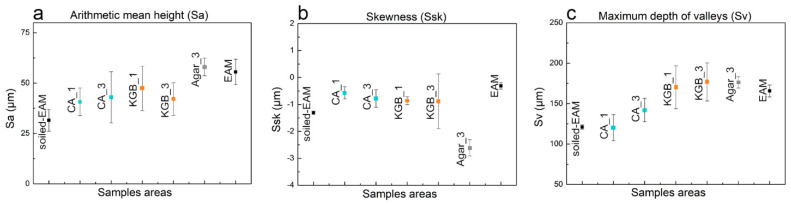
Considered roughness values of Sa (**a**), Ssk (**b**), and Sv (**c**) on the different areas of EAM and soiled−EAM cleaned by different gels and application time CA_1, CA_3, KGB_1, KGB_3, and Agar_3.

Different from WM, the surface of the EAM is visibly rough and inhomogeneous (Figure S4c). The 3D profilometric maps (Figure 7a) and the stereomicroscope observation (Figure 8 and Figure S3) of the surface after the application of the soiling deposits revealed that the particles largely filled the paulownia wood pores. Due to the pronounced roughness, the surface morphology investigation was not fully able to highlight the different cleaning efficacies obtained by CA (Figure 7b), KGB (Figure 7c and Figure S4d), and Agar (Figure S4e). However, relevant information can still be obtained through the surface roughness parameters (Figure 9), which demonstrated the effectiveness of the tested gels—including Agar—for soiling removal. Even if the surface appears morphologically inhomogeneous, we considered the micro-scale differences as markers of the cleaning efficacy: for soiled-EAM, Sa (Figure 9a), and Ssk (Figure 9b) appeared to have similar trends, with CA and KGB included in the same range of values from 1 to 3 applications. On the contrary, Sv (Figure 9c) highlighted some differences: the increase in Sv value in CA_3 and KGB_3 suggested that the soiling deposits were removed better by subsequent applications. In detail, KGB_3 revealed the highest Sv values, comparable with Agar and unsoiled EAM. This result is in accordance with Section 3.2, in which the cleaning efficacy of KGB_3 and Agar_3 revealed better effectiveness compared with CA_3 (Figure 3a).

It is worth noting that the Sv value for EAM is higher with increasing cleaning applications, while for WM the value decreases (Figure 9c). This could be related to the roughness of EAM and to the fact that removing the soil deposits within the wood irregularities uncovers the original high surface roughness, unlike in WM where cleaning makes the surface smooth. The ability of KGB gel to remove in depth the soiling particles from rough areas may be related to its softness and elasticity, as presented in Section 3.1 (Figure 2c,d).

**Figure 10:**
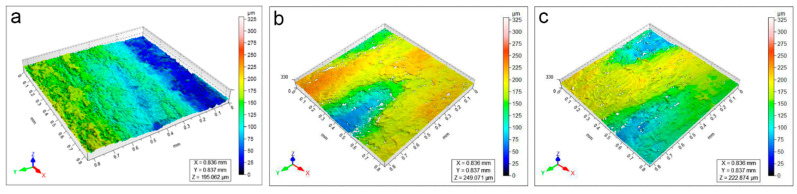
3D maps of areas on sweat-EAM (**a**) cleaned by KGB_1 (**b**) and KGB_3 (**c**). The colour range represents the different heights of the details present on the sample surface; microns are represented on the relative colour scale.

**Figure 11:**
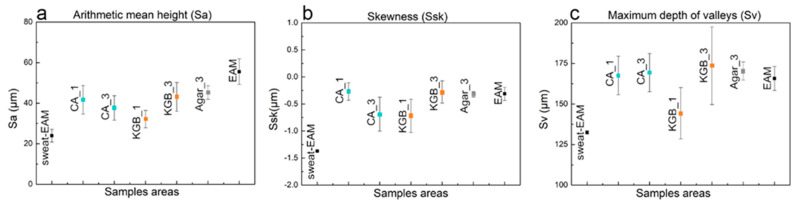
Considered roughness values of Sa (**a**), Ssk (**b**), and Sv (**c**) on the area of EAM and sweat−EAM cleaned by different gels and application time CA_1, CA_3, KGB_1, KGB_3, and Agar_3.

The complex surface morphology of EAM was also found in the sample contaminated with sweat (Figure 10), where a large part of the deposition was penetrated inside the wood pores. By observing the cleaning efficacy through Sa and Ssk (Figure 1a,b), both CA and KGB showed similar values: CA and KGB revealed only slight changes from 1 to 3 applications. The Sv parameter, as for the other considered mock-ups, highlights the greater variations as a result of successive gel applications (Figure 11c), in particular for KGB_1 to KGB_3. By comparing these results with those obtained in the area cleaned with Agar_3 (Figure S4f), no significant difference was revealed, with Agar_3 values comparable with KGB_3 (Figure 11c).

## 4. Conclusions

In this paper, we introduce two biopolymer gels for cleaning, calcium alginate (CA) and konjac glucomannan borax (KGB) gels, loaded with an ecological surfactant for the removal of soiling and sweat deposits from smooth or rough wooden surfaces. These biocompatible products have shown successful performance for artefact conservation, with favourable mechanical properties compared to the traditional Agar rigid-gel.

Compressive and tensile tests were performed on both CA and KGB fresh and aged gels to measure the stability of the mechanical properties over time. CA showed sufficient elasticity and mechanical resistance to be applied to and removed easily from the mock-up surface without breaking. Despite the decrease in this property after ageing, CA proved to be still less brittle than Agar, reducing the risk of leaving residues on the surface after cleaning. KGB gel showed the highest softness and elasticity even after 60 days of storage, which is an advantageous aspect for conservation interventions. The texture of this gel is its most distinctive feature, also compared with Agar, which is instead classified as a rigid gel.

These desirable mechanical characteristics led CA and KGB gel to be effective in the cleaning of different surfaces, including a smooth varnished surface inspired by classical Western bowed string instruments (WM) and a rough and irregular surface treated with the traditional East Asian Nakdong technique (EAM). The monitoring of the soil deposit removal by XRF, in accordance with the morphological profilometric analysis of the cleaned surfaces, demonstrated that KGB was better at cleaning soil from both smooth and rough surfaces. In the WM, efficiency significantly increased after the second application of the gel, while in the EAM, effectiveness reaches good levels of cleaning after just one application. CA showed slightly lower efficiency than KGB on soiled-WM, but comparable on EAM. As for the soiled-WM and -EAM, the control tests performed with Agar revealed results similar to those obtained with CA and KGB, in particular after three cleaning repetitions. Conversely, the cleaning of sweat from both kinds of mock-ups was comparable for all three gels, already after one application.

This research proved evident progress in the cleaning of artefacts by applying CA and KGB on smooth and rough wooden surfaces, implying that the proposed biopolymer gels are able to achieve cleaning performance comparable to the diffuse Agar gel, with fewer application times, and with improved mechanical properties. However, further investigation and application on different materials and surface treatments, as well as on significant real case studies, will be pursued to extend the potential of the application of these gels in the field of cultural heritage cleaning.

## Figures and Tables

**Table 1:** Mechanical properties of gels determined by tensile testing (mean value ± s.d.; *n* = 3). ANOVA one-way, Multiple Range Test (*p* ≤ 0.05): different symbols indicate statistically different data. AUC: Area Under the stress–strain curve.

Classification		Tensile Strength (kPa)	Elongation at Break (%)	AUC
Agar gel	Fresh	13.4 ± 0.1 *	11.8 ± 0.1 °	-
	Aged	-	-	-
CA gel	Fresh	328 ± 59 **	194 ± 17 ^#^	10029 ± 1751 ^§^
	Aged	239 ± 24 **	27 ± 1 ^#^	2396 ± 250 ^§§^

**Table 2:** Mechanical properties of gels determined by tensile testing (mean value ± s.d.; *n* = 3). ANOVA one-way (*p* < 0.05), Multiple Range Test: different symbols indicate statistically different data. AUC: Area Under the stress–strain curve.

Classification		Hardness (kPa)	Young’s Modulus (kPa)	AUC
Agar gel	Fresh	47 ± 2 *	556 ± 47 °	2361 ± 202 ^§^
	Aged	52 ± 2 *	464 ± 96 °	2179 ± 194 ^§^
KGB gel	Fresh	30 ± 3 **	47 ± 0.1 ^#^	766 ± 15 ^§§^
	Aged	40 ± 13 **	30 ± 2 ^#^	868 ± 264 ^§§^

## Data Availability

Data are contained within the article.

## Supplementary Materials

The following supporting information can be downloaded at: https://www.mdpi.com/article/10.3390/polym15010036/s1, Figure S1: Flow chart of preparation of CA and KGB gel; Figure S2: Schematic image of texture analyzer; Figure S3: Stereomicroscopy image of control and are cleaned with Agar; Figure S4: 3D heat map of soiled mock-ups.
