# Supplementary material for: Biopolymer Gels as a Cleaning System for Differently Featured Wooden Surfaces
Source: Polymers (Basel). 2022 Dec 22;15(1):36. doi: 10.3390/polym15010036 (PMC9823702; doi:10.3390/polym15010036)
Supplement: Supplementary file 1 [file polymers-15-00036-s001.zip › polymers-2023908-supplementary.pdf]

SUPPLEMENTARY INFORMATION

Biopolymer Gels as a Cleaning System for Differently Featured Wooden Surfaces

Chaehoon Lee [1,2], Francesca Di Turo [3], Barbara Vigani [4], Maduka L. Weththimuni [1], Silvia Rossi [4], Fabio Beltram [3], Pasqualantonio Pingue [3], Maurizio Licchelli [1], Marco Malagodi [2,5,*], Giacomo Fiocco [2,5,*] and Francesca Volpi [2,5]

[1] Department of Chemistry, University of Pavia, 27100 Pavia, Italy;
[2] Arvedi Laboratory of Non-Invasive Diagnostics, CISRiC, University of Pavia, 26100 Cremona, Italy;
[3] National Enterprise for nanoScience and nanoTechnology (NEST), Scuola Normale Superiore, Piazza dei Cavalieri 7, 56126 Pisa, Italy
[4] Department of Drug Sciences, University of Pavia, 27100 Pavia, Italy
[5] Department of Musicology and Cultural Heritage, University of Pavia, 26100 Cremona, Italy
* Correspondence: marco.malagodi@unipv.it (M.M.); giacomo.fiocco@unipv.it (G.F.)

TABLE OF CONTENTS

- Figure S1: Flow chart of preparation of CA and KGB gel
- Figure S2: Schematic image of texture analyzer
- Figure S3: Stereomicroscopy image of control and are cleaned with Agar
- Figure S4: 3D heat map of soiled mock-ups

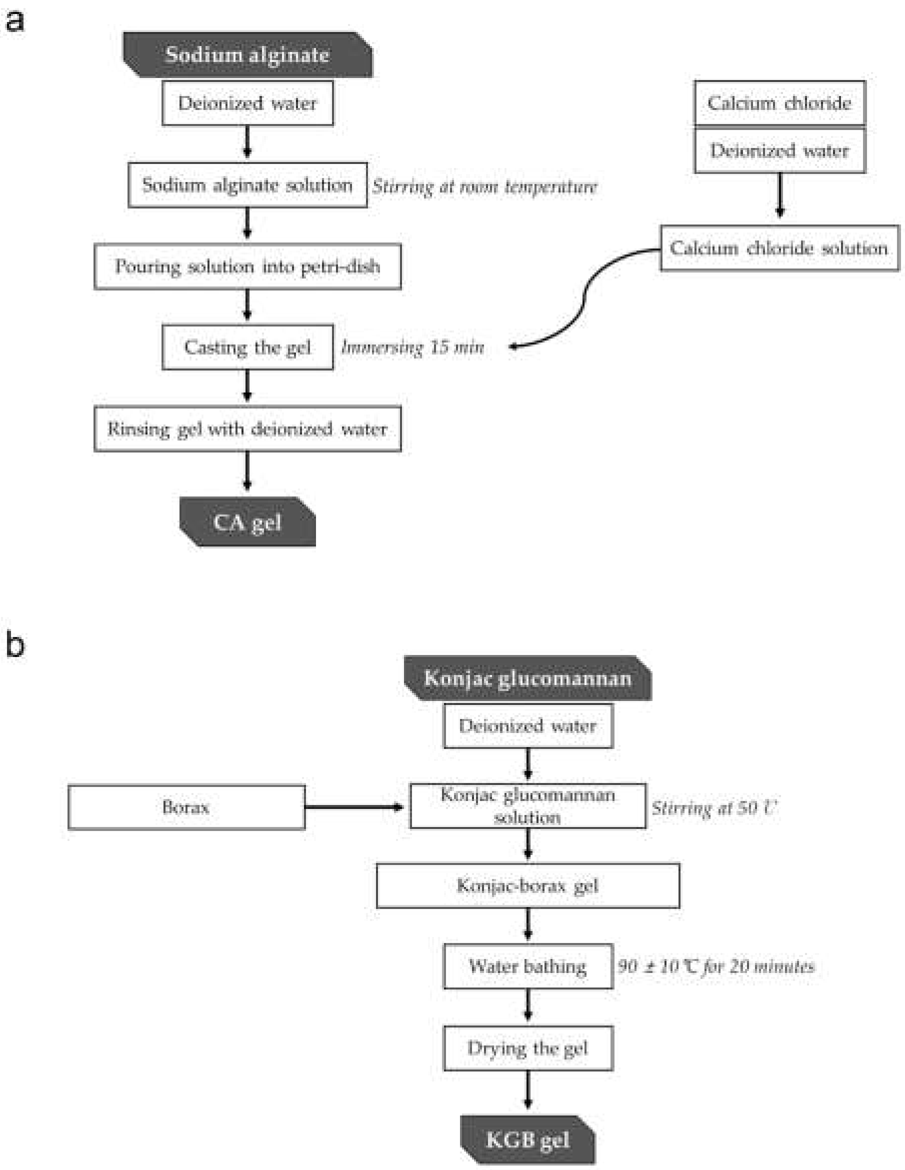

Figure S1. Flow chart for preparation of CA gel by sodium alginate (a) and KGB gel by konjac glucomannan (b).

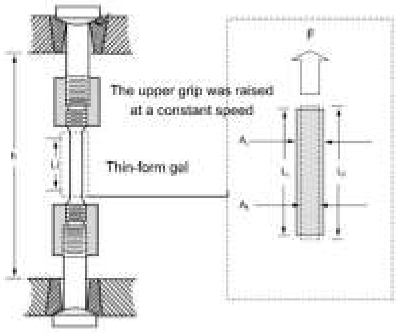

Figure S2. Schematic representation of a tensile test performed using a texture analyzer.

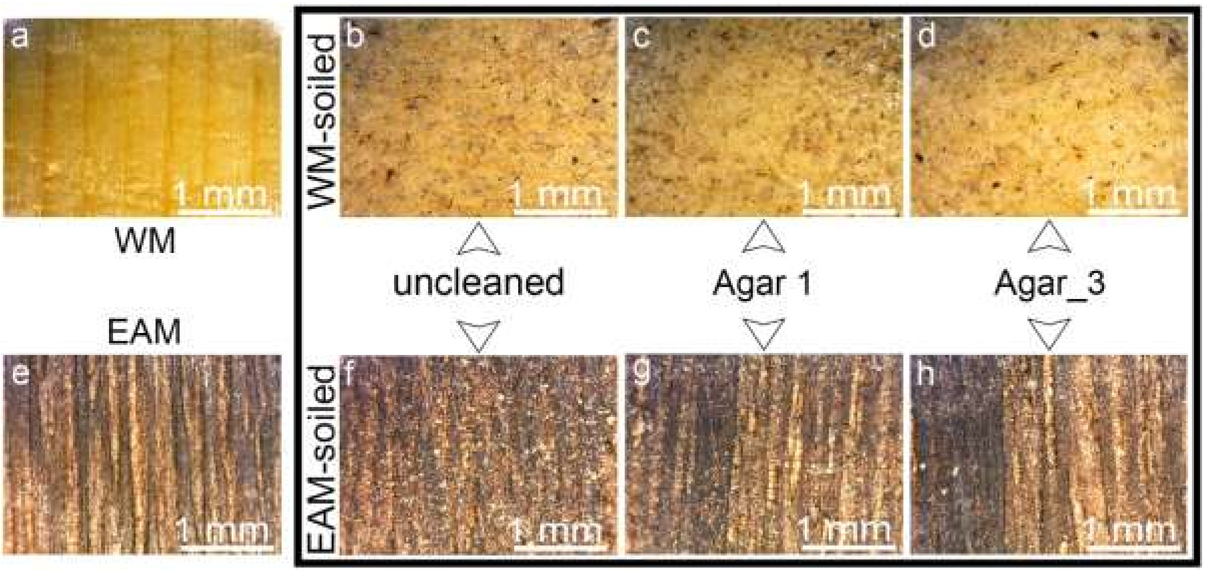

Figure S3. Images by stereomicroscope of the WM (a), a soiled-WM uncleaned area (b), and the areas cleaned by Agar gel at different application times Agar_1 (c), Agar_3 (d); images by stereomicroscope of the EAM (e), a soiled-EAM uncleaned area (f), and the areas cleaned by Agar gel at different application times Agar_1 (g), Agar_3 (h).

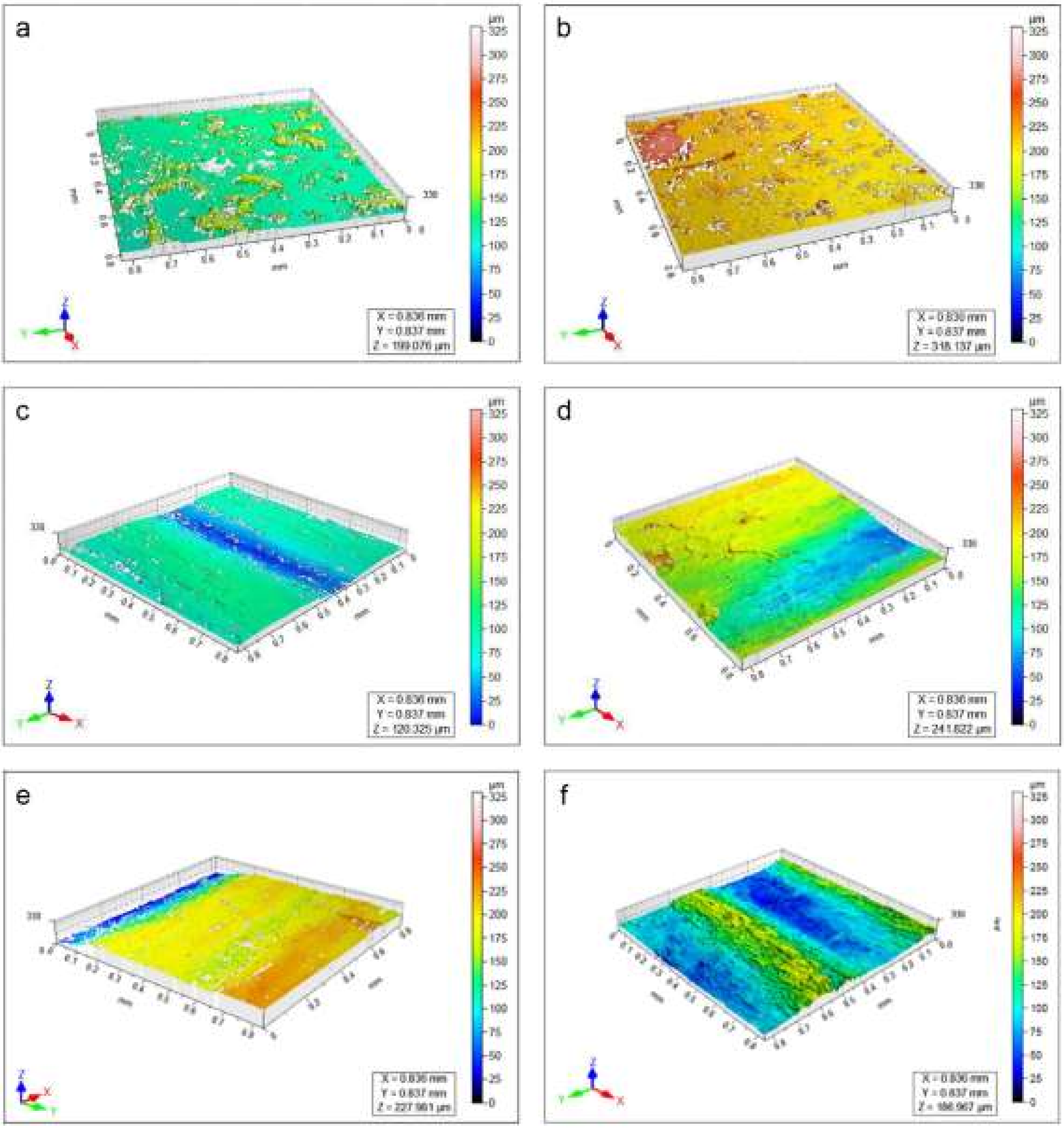

Figure S4. 3D heat maps: soiled-WM cleaned by CA_1 (a) and KGB_1 (b); EAM (c) and soiled-EAM cleaned by KGB_1 (d) and Agar_3 (e); sweat-EAM cleaned by Agar_3 (f).
